# Biologic Responses to House Dust Mite Exposure in the Environmental Exposure Unit

**DOI:** 10.3389/falgy.2021.807208

**Published:** 2022-01-07

**Authors:** Lubnaa Hossenbaccus, Sophia Linton, Jenny Thiele, Lisa Steacy, Terry Walker, Crystal Malone, Anne K. Ellis

**Affiliations:** ^1^Department of Medicine, Queen's University, Kingston, ON, Canada; ^2^Allergy Research Unit, Kingston Health Sciences Centre–KGH Site, Kingston, ON, Canada; ^3^Department of Biomedical and Molecular Sciences, Queen's University, Kingston, ON, Canada

**Keywords:** allergic rhinitis, Environmental Exposure Unit, house dust mite, cytokines, skin prick test, immunoglobulin E, Der p, Der f

## Abstract

**Introduction:** Allergic rhinitis (AR) is an inflammatory disease of the nasal mucosa that can be modeled using Controlled Allergen Exposure Facilities (CACF). Recently, we clinically validated the house dust mite (HDM) Environmental Exposure Unit (EEU) facility. In the current study, we aimed to assess biological responses in the blood following HDM exposure in the HDM-EEU.

**Methods:** Fifty-five participants passed a screening visit, where they provided consent and completed a skin prick test (SPT), then attended a modest or higher HDM exposure session. Baseline and post-exposure blood samples were collected. Complete blood counts with differentials were measured, and isolated serum was used to determine *Dermatophagoides farinae*- and *Dermatophagoides pteronyssinus*-specific IgE (sIgE) and cytokine concentrations (IL-4, IL-5, IL-6, IL-10, IL-13, TNF-α).

**Results:** HDM-allergic participants had significantly greater SPT wheal sizes than healthy controls. sIgE concentrations were significantly greater in allergic participants, with a strong correlation between *Dermatophagoides farinae* and *Dermatophagoides pteronyssinus*. Serum eosinophil counts were significantly decreased post-exposure for allergic participants. White blood cell, neutrophil, and lymphocyte counts were significantly increased for both allergic and non-allergic participants post-exposure. Serum IL-13 concentrations were significantly reduced post-exposure in allergics while TNF-α was significantly reduced in non-allergics.

**Conclusion:** The HDM-EEU is a useful model for investigating biologic mechanisms of HDM-induced AR. Allergic participants produced measurable biological changes compared to healthy controls following allergen exposure, specifically with serum expression of eosinophils and related markers, namely IL-5, which promotes the proliferation and differentiation of eosinophils, and IL-13, a cytokine released by eosinophils. The exact mechanisms at play require further investigation.

## Introduction

Allergic rhinitis (AR) is a nasal inflammatory disease triggered by exposure to seasonal or perennial allergens, such as animal dander and house dust mite (HDM), which are present year-round. Common species include the American (*Dermatophagoides farinae*) and European (*Dermatophagoides pteronyssinus*) HDMs, and the prevalence of sensitization to these mites is reported to be up to 90% in various countries (1). Over 35 allergens have been isolated from the feces of HDM, with Der p 1, Der p 2, Der f 1, and Der f 2 being the main culprits in the induction of AR symptoms (2). Diagnosis of an HDM allergy involves a review of clinical history and physical examination as well as diagnostic testing including skin prick testing (SPT) and HDM-specific IgE testing.

Allergic sensitization involves the processing of allergens by antigen presenting cells, such as dendritic cells, and presentation on major histocompatibility complex (MHC) class II molecules on the cell surface. Through the T cell receptor (TCR), naïve T cells are primed and differentiate to type 2 helper (Th2) T cells, elucidating a predominantly Th2-mediated immune response (3). Cytokines released by Th2 cells, such as interleukin (IL)-4, IL-5, and IL-13, stimulate IgE production and class-switching and encourage the differentiation of eosinophils to promote allergic inflammation (4–6).

Following re-exposure, allergens crosslink to cell-bound IgE on mucosal mast cells, resulting in degranulation and the release of pre-formed cytoplasmic inflammatory molecules such as (but not limited to) histamine (7). The release of these molecules characterizes the early phase AR response, occurring in a matter of minutes and resulting in the clinical symptoms of nasal itching, sneezing, and rhinorrhea, through increased vascular permeability and mucous secretion.

Mast cells also contribute to the late phase AR response, occurring between 4 and 8 h following allergen exposure (8). Cytokines further promote the recruitment of other inflammatory mediators and cells from the peripheral blood to the nasal mucosa (9, 10). As a result, the nasal mucosa is primed for further allergen exposure and causes persistent symptoms including nasal obstruction or congestion. These late phase inflammatory processes affect tissue remodeling.

Allergic rhinitis can be modeled using Controlled Allergen Challenge Facilities (CACFs). These facilities are custom designed and specifically engineered to control variables including, but not limited to, air quality, temperature, humidity, allergen type, and most importantly allergen concentration with a large group of participants. In addition to highly accurate and complete symptom reporting, CACFs also permit the collection of biologic samples, including blood and nasal specimens. These may provide insights into serum cytokine concentrations throughout or following allergen exposure and genetic or epigenetic changes (11, 12).

The Environmental Exposure Unit (EEU) was the first CACF to be built in North America. Established in the late 1980's, and currently located at the Kingston Health Sciences Centre–KGH site, the EEU has been used extensively for the evaluation of ragweed, grass, and birch allergy (13–16). A specially designed facility now housed within the main EEU was developed to study perennial allergens, the HDM-EEU, and can host 5 to 35 participants per session.

In August 2019, we clinically validated the HDM-EEU, demonstrating that it can generate AR symptoms in HDM-allergic individuals (17). We exposed participants to a modest [(Der f 1) = 2.67 ng/m^3^ and (Der p 1) = 2.07 ng/m^3^] or higher HDM [(Der f 1) = 3.80 ng/m^3^ and (Der p 1) = 6.66 ng/m^3^] target for 3 h and measured symptoms for up to 24 h post-exposure. Allergic participants exposed to a higher HDM target experienced a significantly greater peak in mean TNSS at 2.5 (*p* < 0.05) and 3 h (*p* < 0.01) compared to modest target allergics. Compared to healthy controls, allergics experienced significantly elevated TNSS and TRSS from 1 to 5 h following the onset of allergen exposure, irrespective of allergen concentration. Blood samples were collected pre- and post-HDM exposure using the HDM-EEU and here we report the biologic responses of HDM-allergic and non-allergic participants.

## Materials and Methods

### Study Design

**Figure 1:**
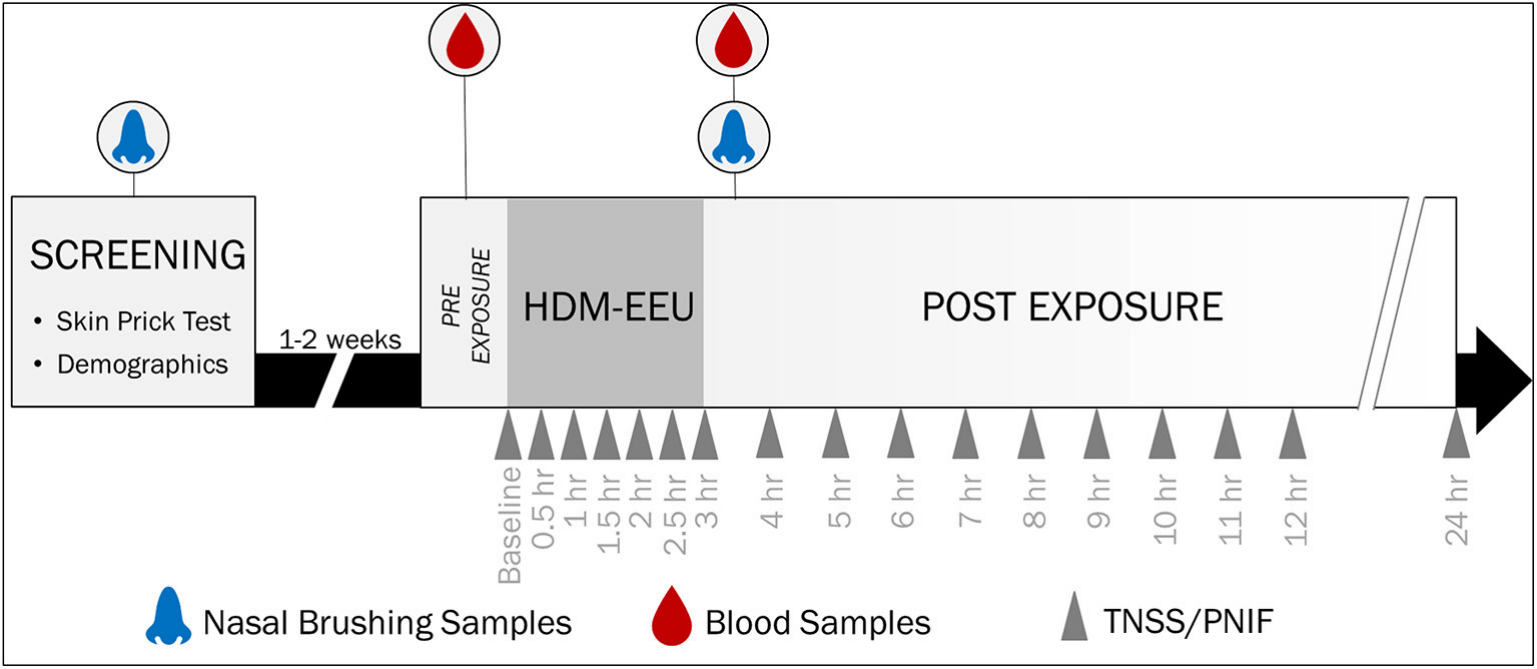
Study Design and Sample Collection. Participants were recruited for a screening visit, where skin prick testing was completed to evaluate allergic sensitization to a panel of allergens. Eligible participants were invited to a modest or higher 3-h HDM exposure session in the HDM-EEU. Blood and nasal samples were collected pre- and post-HDM exposure. Hossenbaccus et al. (17).

Participant recruitment and study inclusion/exclusion criteria for this study were previously published (17). In short, sixty-eight participants 12 to 65 years of age were recruited and attended a screening visit where SPT was completed (Figure 1). Fifty-five participants passed screening, with forty-four HDM-allergics and eleven non-allergic controls, who were not sensitized to any allergen evaluated on the SPT panel. Thirty eligible participants attended a modest and twenty-five attended a higher HDM exposure session in the HDM-EEU. Blood and nasal samples were collected before and after HDM exposure. Peripheral blood collected in PAXgene Blood RNA tubes (PreAnalytiX) pre-exposure are reported elsewhere (18) and nasal sample findings are not reported here.

### Skin Prick Testing

SPT was performed at screening on the volar surface of the participant's forearm using allergen extracts prepared in a Duotip-Test® II Dipwell tray. The allergen panel included *D. pteronyssinus* [ALK-Abelló; 10,000 allergy units (AU)/mL], *D. farinae* (ALK-Abelló; 10,000 AU/mL), Timothy grass [ALK-Abelló; 100,000 bioequivalent allergy units (BUA)/mL], ragweed [ALK-Abelló; weight per volume (w/v) 1:20], birch (ALK-Abelló; w/v 1:20), cat (Hollister-Stier; 10,000 BAU/mL), dog (ALK-Abelló; w/v 1:20), oak (ALK-Abelló; w/v 1:20), alder (ALK-Abelló; w/v 1:20), and *Alternaria* (ALK-Abelló; w/v 1:20). Histamine and glycerin phenol-saline were the positive and negative controls, respectively. The panel was administered using sterile plastic bifurcated Duotip-Test II devices. Results for *D. pteronyssinus* and *D. farinae* were determined to be positive if the wheal diameter was 5 mm of greater than the negative control. For all other allergen extracts, a positive result was classified as a wheal diameter of 3 mm or greater than the negative control.

### Complete Blood Count With Differential

Hematology samples collected in EDTA tubes (BD) were used for complete blood count (CBC) with differential analysis, processed by the Kingston Health Sciences Centre–KGH site Core Laboratory.

### Serum Samples

Blood samples were collected pre- and post-exposure, consisting of a serum separator tube (SST, BD).The SST tubes were allowed to clot at room temperature for 30 min (19). The clot was separated from the serum following the centrifugation of the tubes at 1,500 g for 15 min at room temperature. The serum was aliquoted into 3 microfuge tubes (Sarstedt) using a 1,000 μL pipette, such that each contained ~500 μL of sample. The tubes were frozen and stored at −80°C.

### Serum HDM-Specific IgE

Frozen serum samples were thawed and 400 μL was aliquoted into test tubes. The Phadia^TM^ 100 and ImmunoCAP® assay (Somagen^TM^ Diagnostics) were used to measure the concentration of *D. pteronyssinus* and *D. farinae*-specific IgE. Calibrators (0.001, 0.35, 0.70, 3.50, 17.5, and 100 kUA/L), two curve controls, quality controls (low, medium, and high), a negative control, and positive internal controls were used. The fluorescence of the eluate was measured to determine the concentration of sIgE in the samples, relative to a calibration curve established in the first run. Two curve controls were used in subsequent assays against the same calibration curve. The assay procedures were all completed by the instrument.

### Serum Cytokine Concentrations

Frozen pre- and post-exposure serum samples were thawed, and the following cytokines were evaluated using a Human High Sensitivity T Cell Magnetic Bead Panel assay: IL-4, IL-5, IL-6, IL-10, IL-13, and TNF-α. The assay was performed as per the manufacturer's protocol. The plate was run on the Bio-Plex® 200^TM^ using the Bio-Plex Manager 6 software.

### Statistical Analysis

Pre- and post-exposure mean cell counts (x10^9^/L) and cytokine concentrations were plotted for allergics and non-allergics, analyzed using Wilcoxon matched-pairs signed rank tests. Change in mean cell counts and cytokine concentrations for allergics and non-allergics, as well as when stratified by HDM exposure level (modest vs. higher) were evaluated using Mann-Whitney tests. Percent eosinophil counts relative to white blood cell concentrations and sIgE concentrations were analyzed using a Kruskal-Wallis test with Dunn's multiple comparisons. Correlations were evaluated using Pearson correlation coefficients. GraphPad Prism 9.2.0 software was used for analysis and graphing.

## Results

### Skin Prick Test Findings

Fifty-five participants successfully completed this study, with twenty-four allergics and six non-allergics attending the modest HDM allergen target concentration session and twenty allergics and five non-allergics attending the higher HDM target concentration session. Most allergic participants were polysensitized to various allergens evaluated using SPT, with only 4 participants who were monosensitized to just the two HDM allergen extracts (Table 1).

**Table 1:** SPT Sensitization.

**Allergen**	**Number of allergic participants**
*D. pteronyssinus*	44
*D. farinae*	44
Timothy Grass	22
Ragweed	32
Birch	25
Cat	22
Dog	7
Oak	9^*^
Alder	12^**^
*Alternaria*	9

* *Out of 25 participants*.

** *Out of 24 participants*.

**Figure 2:**
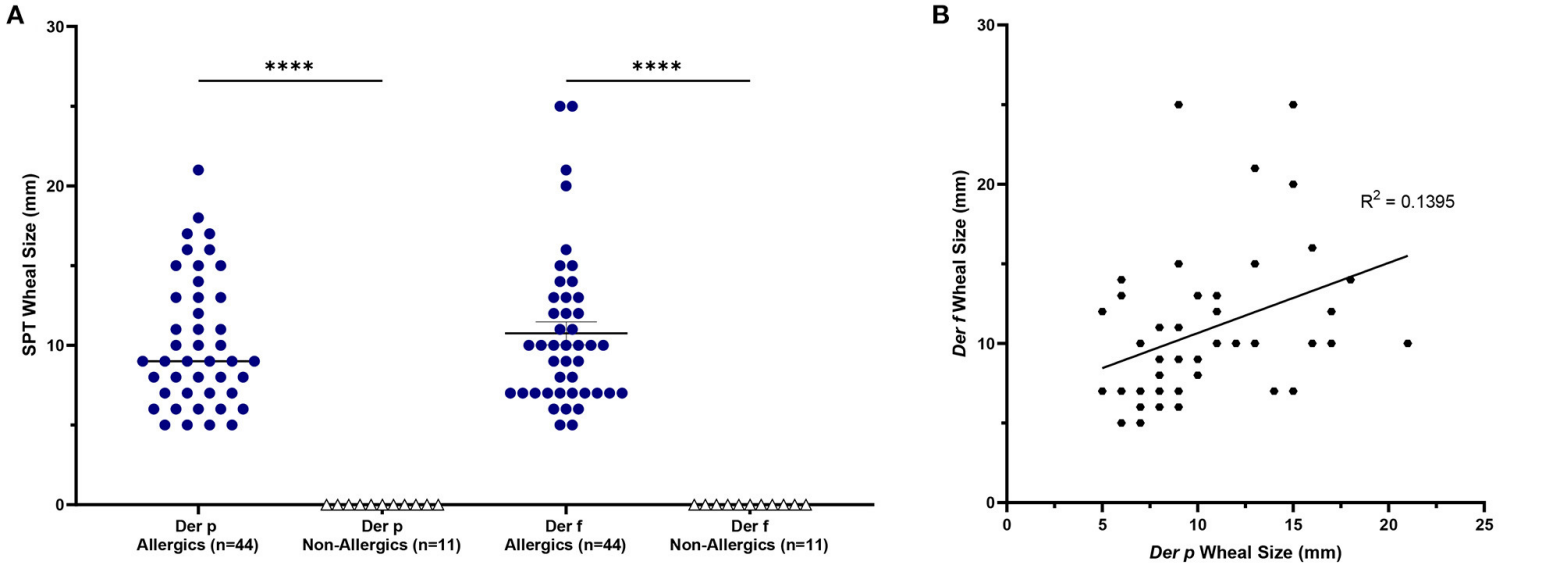
SPT wheal sizes were significantly elevated for allergics. Allergics had significantly elevated SPT wheal sizes for both Der p and Der f compared to non-allergics **(A)**. A weak correlation (R^2^ = 0.1395) was observed between wheal sizes of the two HDM allergens **(B)**. ****, *p* < 0.0001.

HDM-allergics had significantly bigger (*p* <0.0001) SPT wheal diameters for both *D. pteronyssinus* and *D. farinae* extracts than non-allergic controls (Figure 2A). Wheal sizes between the two allergen extracts were poorly correlated (Figure 2B).

Allergic participants showed greater allergic sensitization to HDM than non-allergic controls.

### Complete Blood Counts With Differentials

**Figure 3:**
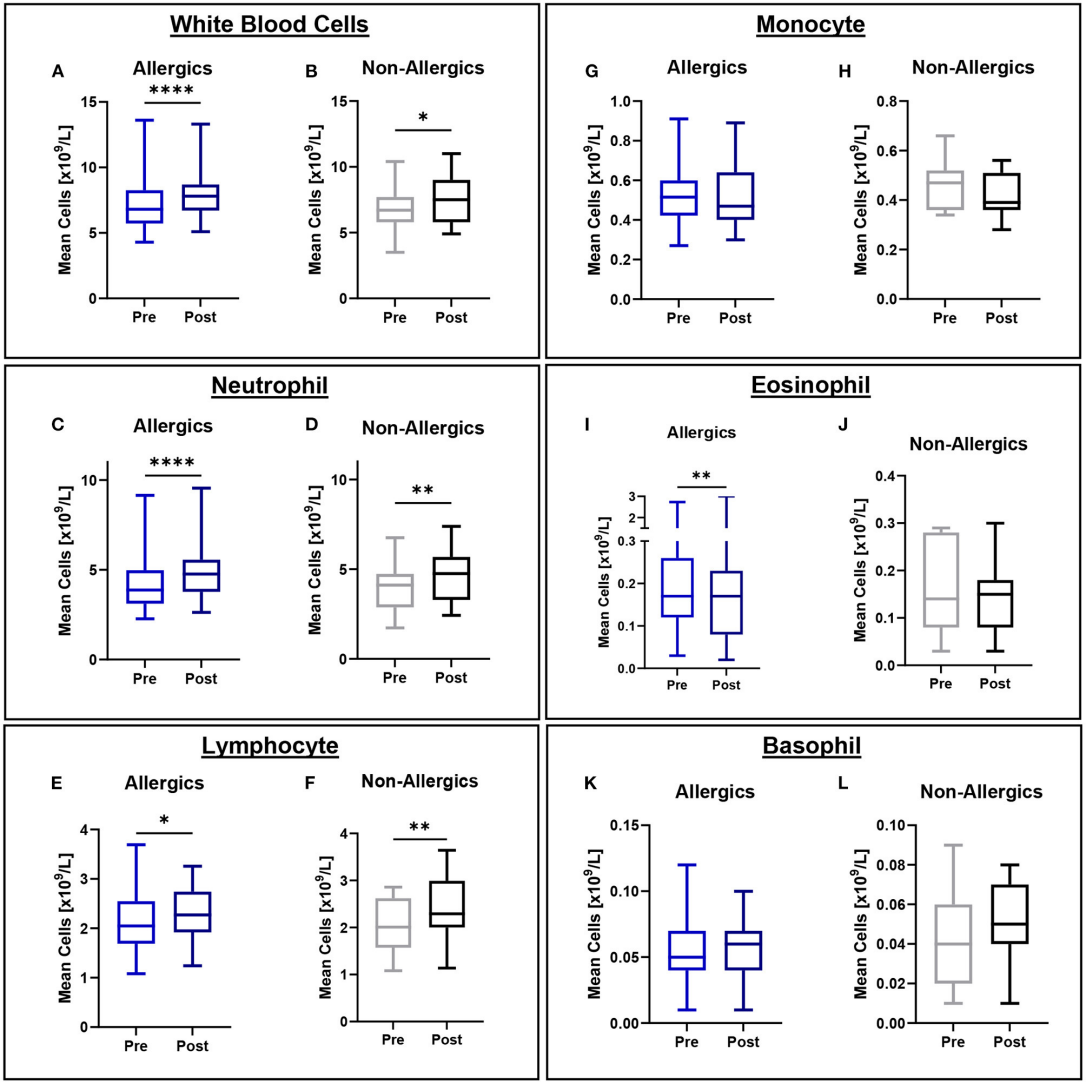
White blood cell counts in peripheral blood collected pre- and post-exposure for HDM-allergic and non-allergic participants. Concentrations of white blood cells **(A,B)**, neutrophils **(C,D)**, lymphocytes **(E,F)**, monocytes **(G,H)**, eosinophils **(I,J)**, and basophils **(K,L)** were evaluated pre- and post-HDM exposure for allergic and non-allergic participants in these paired analyses. Eosinophil concentrations were significantly decreased only for HDM-allergic participants **(I)**. *, p< *0.05*, **, p< *0.01*, ****, p< *0.0001*.

**Figure 4:**
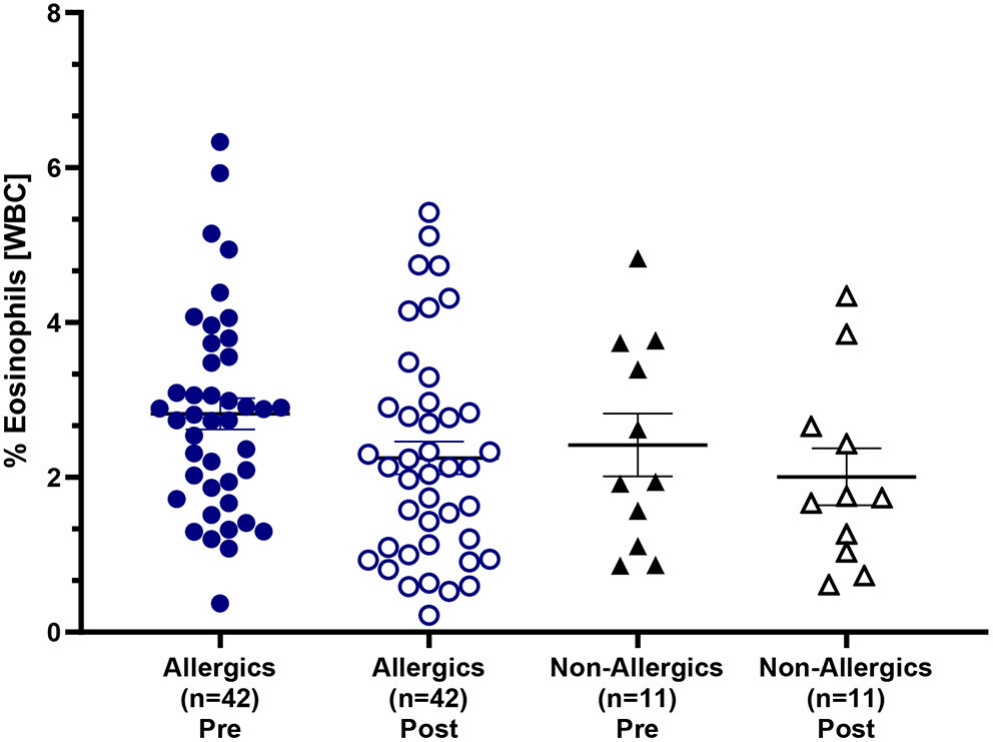
Eosinophil counts as a percentage of white blood cells in the peripheral blood. Blood samples collected pre- and post-HDM exposure were evaluated for complete blood counts with differentials. Eosinophils were evaluated as a percentage of all white blood cells in the peripheral blood. Both allergic and non-allergic participants experienced a decrease in % eosinophils post-exposure compared to pre-exposure.

Mean eosinophil counts were significantly decreased (*p* < 0.01) in the peripheral blood of only allergic participants post-exposure compared to baseline (Figures 3I, J). When evaluating eosinophils as a percentage of all white blood cells, no significant differences were observed (Figure 4).

Mean white blood cell (WBC), neutrophil, and lymphocyte counts were significantly elevated for both allergics (*p* < 0.0001 for WBC; *p* < 0.0001 for neutrophils; p < 0.05 for lymphocytes) and non-allergics (*p* < 0.05 for WBC; *p* < 0.01 for neutrophils; *p* < 0.01 for lymphocytes) post-HDM exposure compared to baseline (Figures 3A–F). Mean monocyte and basophil counts were not significantly different for either non-allergics or allergics (Figures 3G,H,K,L).

**Figure 5:**
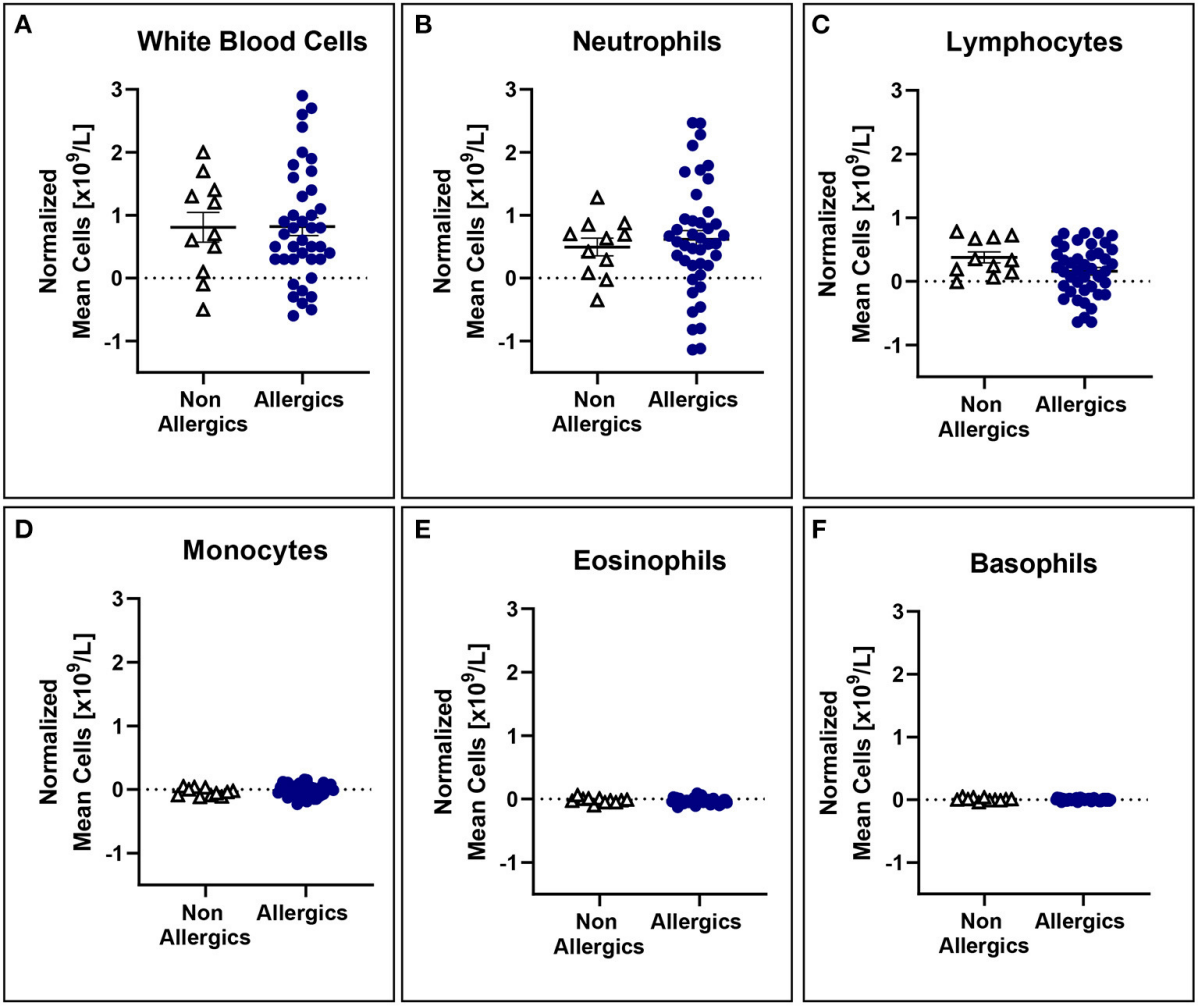
White blood cell counts normalized to baseline are comparable for both allergic and non-allergic participants. No significant differences were observed in white blood cell **(A)**, neutrophil **(B)**, lymphocyte **(C)**, monocyte **(D)**, eosinophil **(E)**, and basophil **(F)** counts normalized to baseline in the peripheral blood.

**Figure 6:**
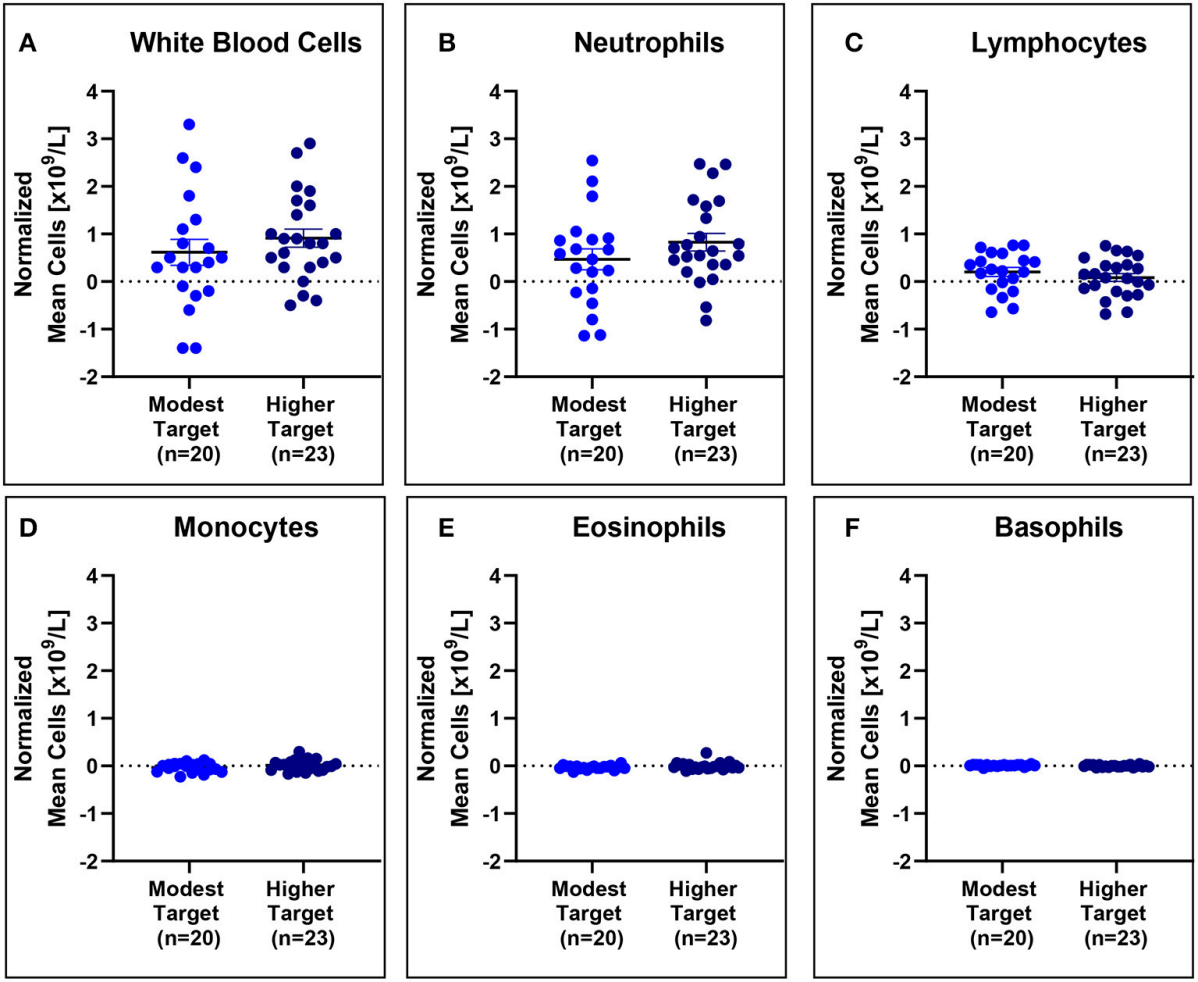
White blood cell counts are comparable for modest and higher target allergics. No significant differences were observed in change in white blood cell **(A)**, neutrophil **(B)**, lymphocyte **(C)**, monocyte **(D)**, eosinophil **(E)**, and basophil **(F)** counts normalized to baseline in the peripheral blood post-exposure for allergics exposed to a higher vs. modest HDM target.

No significant differences in mean white blood cell counts normalized to baseline were observed (Figure 5), even when stratified based on HDM exposure concentration (Figure 6).

Complete blood counts with differential show the occurrence of non-specific inflammation as well as post-exposure changes in eosinophil concentrations in allergic participants.

### Serum HDM-Specific IgE

**Figure 7:**
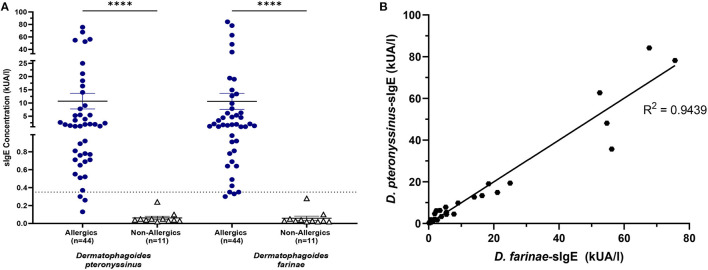
HDM-specific IgE concentrations were significantly increased for allergics. Allergics had significantly elevated *Dermatophagoides pteronyssinus-* and *Dermatophagoides farinae*-sIgE compared to non-allergics **(A)**. Concentrations of *D. pteronyssinus-* and *D. farinae*-sIgE were strongly correlation **(B)**. ****, *p* < 0.0001.

HDM-allergics had significantly greater (*p* < 0.0001) *D. pteronyssinus* and *D. farinae* compared to non-allergic controls (Figure 7A).

**Figure 8:**
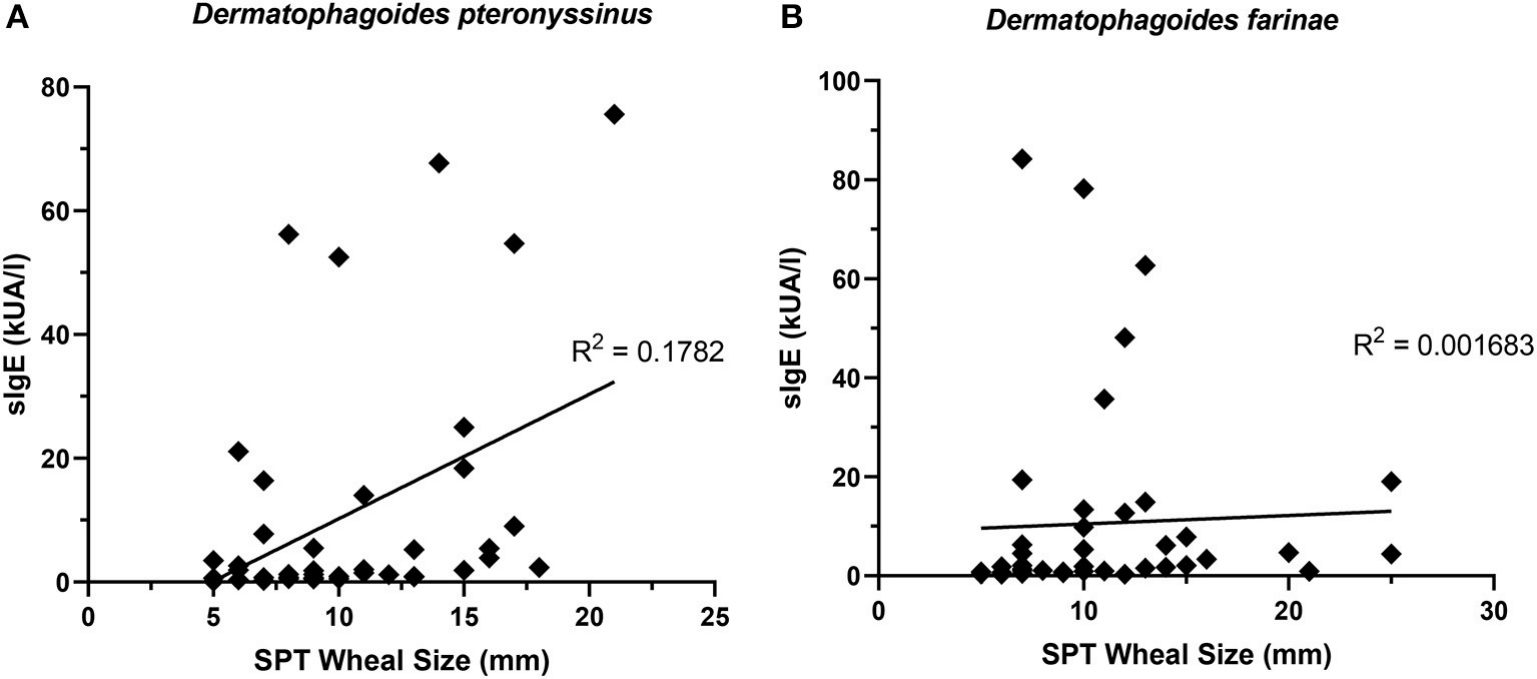
sIgE concentrations and SPT wheal sizes were poorly correlated. SPT wheal sizes and sIgE were poorly correlated for both *Dermatophagoides pteronyssinus* **(A)** R^2^ = 0.1782 and *Dermatophagoides farinae* **(B)** R^2^ = 0.001683.

The presence of one HDM-specific IgE was strongly correlated (R^2^ = 0.9439) with the other (Figure 7B), though poor correlations were observed between SPT wheal sizes and serum sIgE concentrations for *D. pteronyssinus* (Figure 8A) and *D. farinae* (Figure 8B).

### Serum Cytokine Concentrations

**Figure 9:**
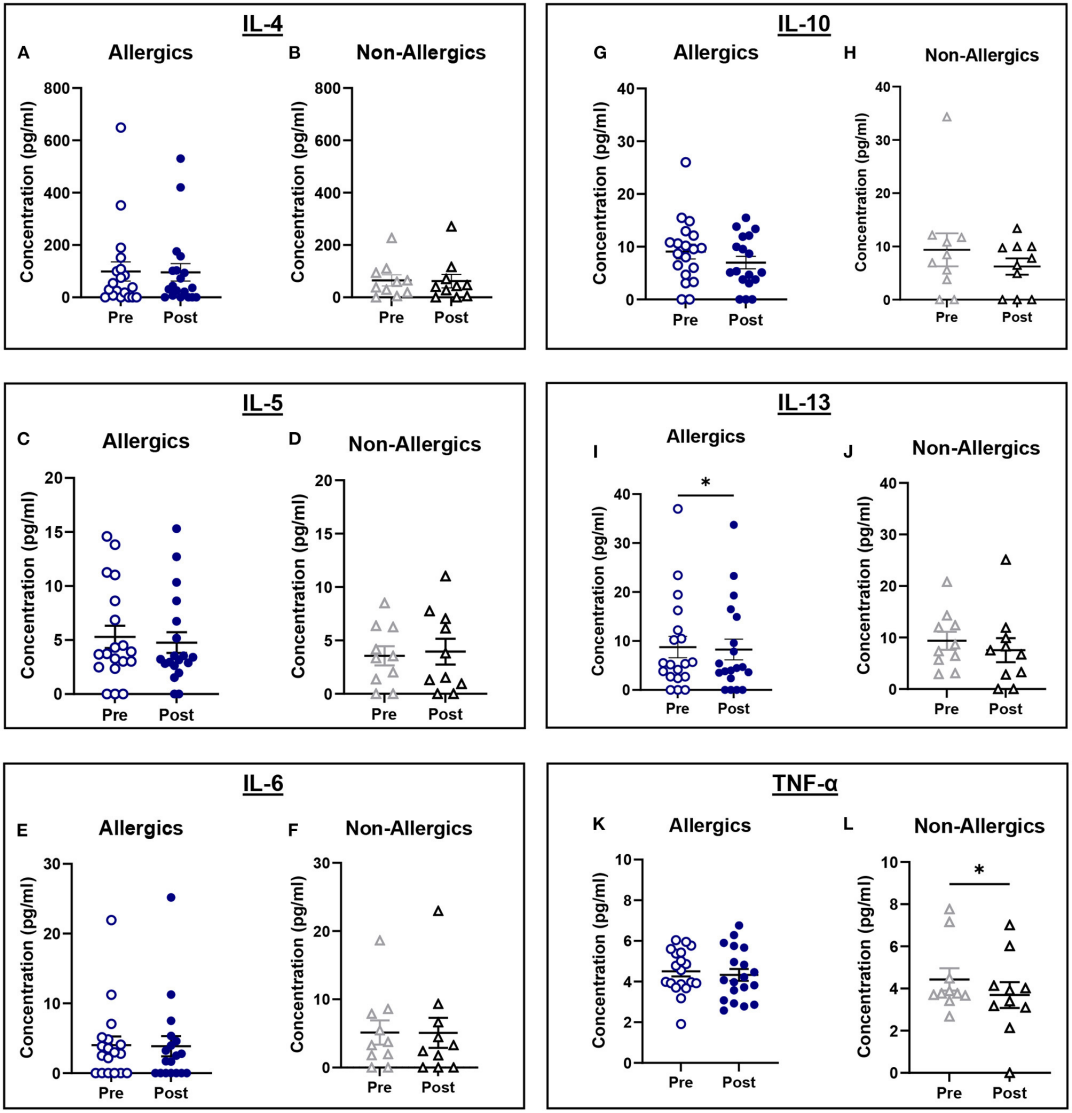
Serum cytokine concentrations collected pre- and post-exposure for HDM-allergic and non-allergic participants. Concentrations of IL-4 **(A,B)**, IL-5 **(C,D)**, IL-6 **(E,F)**, IL-10 **(G,H)**, IL-13 **(I,J)**, and TNF-α **(K,L)** were evaluated pre- and post-HDM exposure for allergic and non-allergic participants in these paired analyses. IL-13 concentrations were significantly decreased only for HDM-allergic participants **(I)**, while TNF-α was significantly decreased in non-allergic participants. *, p< *0.05*.

Serum IL-13 concentrations were significantly reduced (*p* < 0.05) in allergics post-exposure (Figure 9I) while TNF-α was significantly reduced (*p* < 0.05) in non-allergics post-exposure (Figure 9L) in paired analyses. No other significant differences were observed (Figures 9A–H; J, K).

**Figure 10:**
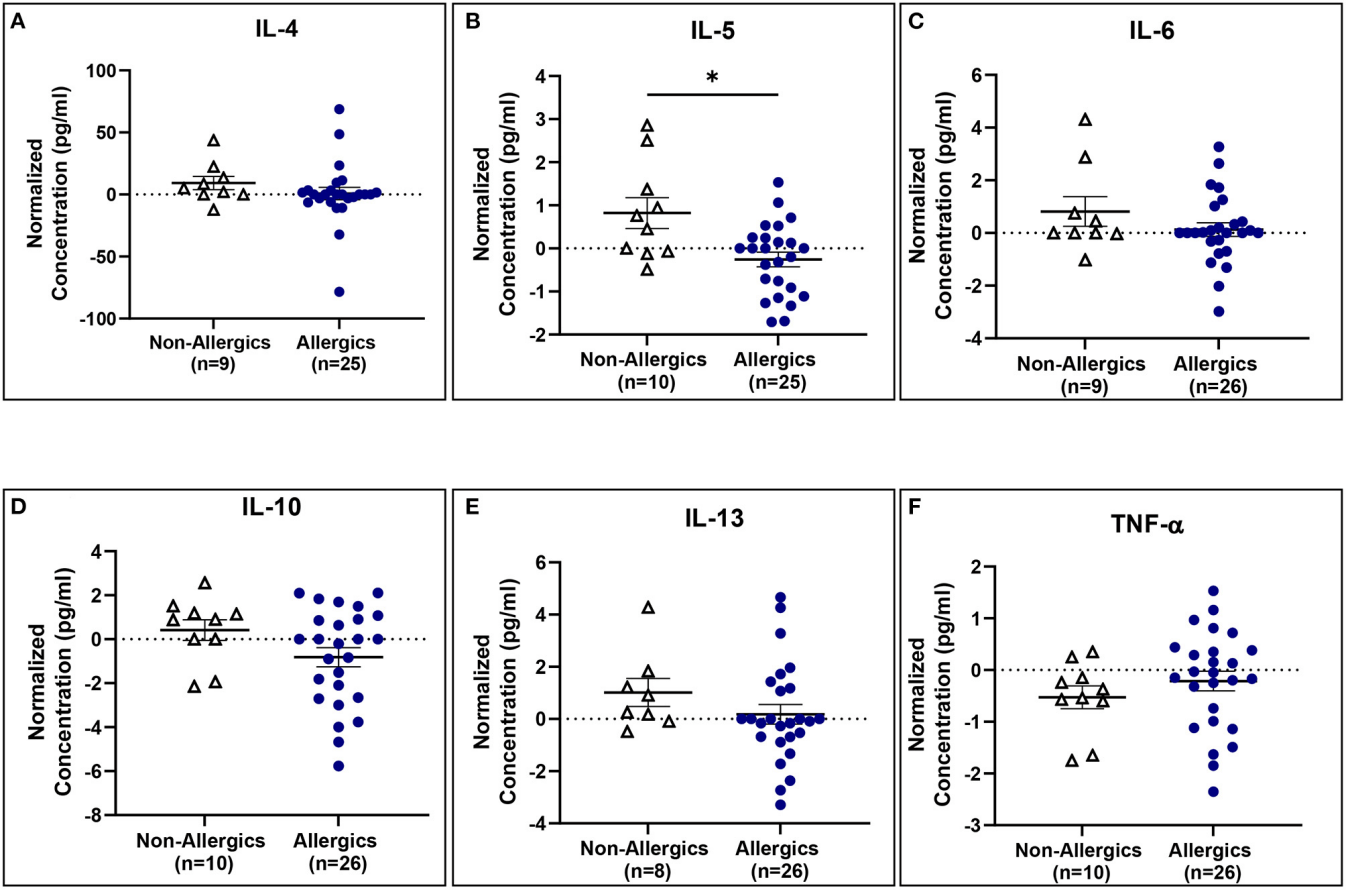
Serum IL-5 concentrations normalized to baseline are significantly reduced for allergics compared to non-allergic participants. Concentrations of IL-4 **(A)**, IL-5 **(B)**, IL-6 **(C)**, IL-10 **(D)**, IL-13 **(E)**, and TNF-α **(F)** normalized to baseline were evaluated pre- and post-HDM exposure for allergic and non-allergic participants. IL-5 concentrations normalized to baseline were significantly decreased for HDM-allergic participants compared to non-allergic controls. *, p< *0.05*.

IL-5 concentrations normalized to baseline were significantly reduced (*p* < 0.05) for allergics compared to healthy controls (Figure 10B), though not for the other cytokines (Figures 10A, C–F).

**Figure 11:**
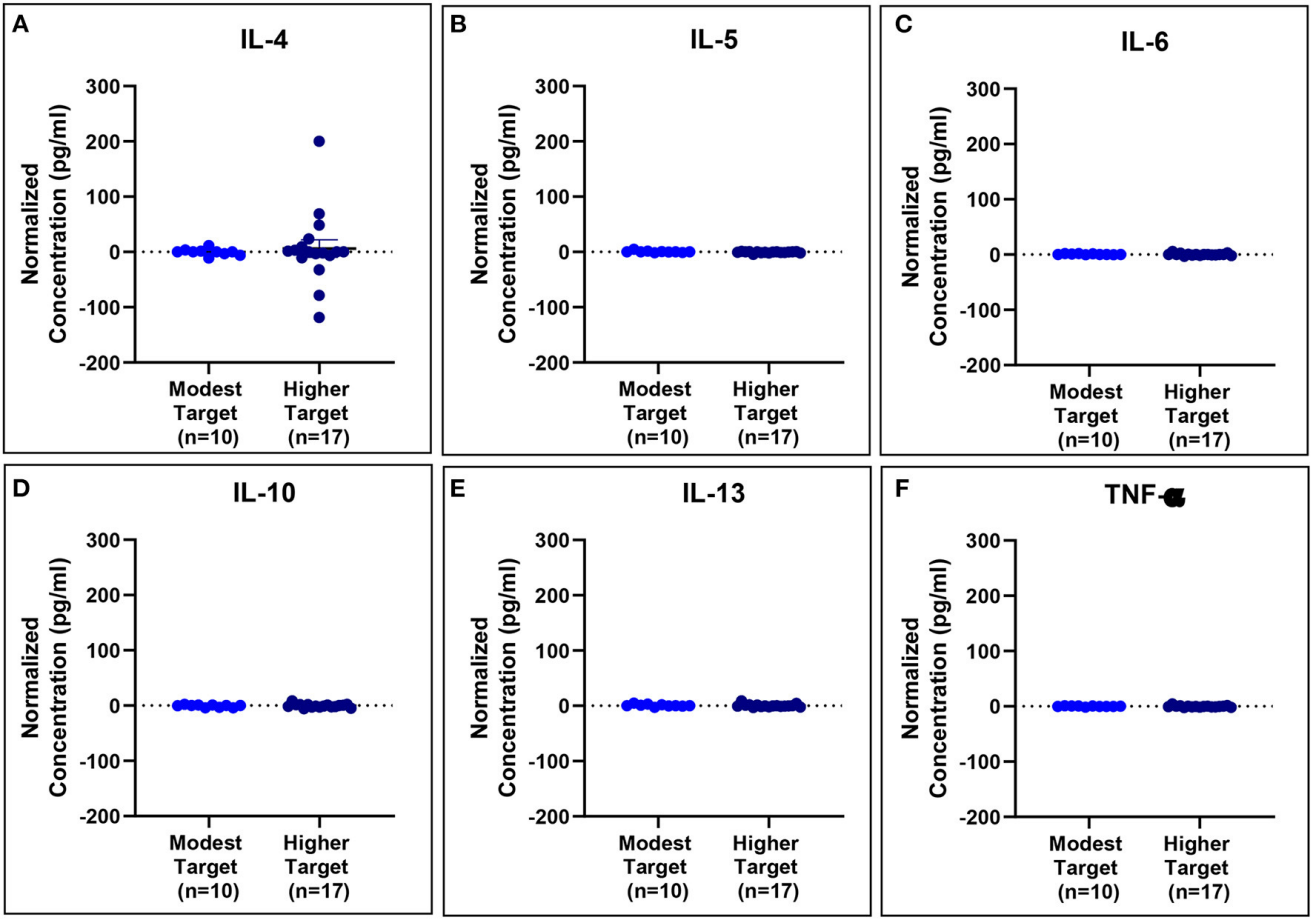
Serum cytokine concentrations normalized to baseline are comparable for modest and higher target allergics. No significant differences were observed in IL-4 **(A)**, IL-5 **(B)**, IL-6 **(C)**, IL-10 **(D)**, IL-13 **(E)**, and TNF-α **(F)** concentrations normalized to baseline in the peripheral blood post-exposure for allergics exposed to a higher vs. modest HDM target.

Serum cytokine concentrations reveal post-exposure changes in concentrations of eosinophil-associated mediators (IL-5 and IL-13) (Figure 11).

## Discussion

HDM-allergic participants had significant changes in biological responses compared to non-allergic controls following HDM exposure in the HDM-EEU.

HDM-allergic participants had significantly elevated and more variable concentrations of *D. pteronyssinus-* and *D. farinae-sIgE* when compared to non-allergic participants, and a strong correlation between the two species. This finding is intriguing as *D. pteronyssinus* is the European HDM, whereas *D. farinae* is the American HDM. Many studies have shown that most North American homes contain measurable levels of both *D. pteronyssinus* and *D. farinae*, and it raises the question as to how the European HDM has become so prevalent in North America (20). In contrast, a European study found a weak correlation between the concentrations of Der p 1 and Der f 1 in homes in two German cities (21). While this is a more epidemiological consideration, it illustrates the effect of travel and globalization on the spread of allergic disease. Additionally, cross-reactivity between the two species may be associated with the strong correlation observed.

sIgE was not well correlated to SPT wheal sizes. While SPT and the sIgE assay are important measures of participant's allergen sensitization and are used diagnostically, they are not interchangeable (22). SPTs reflect a targeted, localized immune response to an allergen *in vivo* and are corrected for skin histamine sensitivity by subtracting the negative control (23). SPT has the clear advantage of having a rapid turn around time, they are relatively inexpensive, and are highly sensitive (22). In our cohort, three symptomatic allergic participants had very low (<0.35 kUA/L) *D. pteronyssinus*-sIgE concentrations, two of whom also had very low *D. farinae*-sIgE concentrations, despite positive SPT results.

Paired analyses of whole blood CBCs for allergic and non-allergic participants revealed significant post-HDM exposure increases in white blood cell, neutrophil, and lymphocyte concentrations. This non-specific inflammation may be associated with small amounts of endotoxin exposure and generalized nasal irritation to the HDM allergens. Allergic participants had significantly decreased eosinophil counts post-exposure, unlike healthy controls, and had elevated percentages, though not significantly so, of eosinophils in the peripheral blood both pre- and post-exposure compared to their non-allergic counterparts. A drop in blood eosinophils may indicate cell migration to the nasal mucosa, as previous findings following Bermuda grass challenge in the NAC revealed significantly increased nasal eosinophil counts from nasal lavage samples (24). Eosinophil cationic protein (ECP), a marker of eosinophil activation, has also been found to be significantly increased by twofold in nasal fluid samples of individuals with perennial AR compared to controls (25).

Paired analyses of pre- and post-exposure serum cytokine concentrations for allergic and non-allergic participants were generally comparable, with all except for IL-5 for non-allergics decreasing post-HDM exposure. The pro-inflammatory cytokines related to Th2 activation (IL-4, IL-5, and IL-13) were expected to increase in allergic participants compared to non-allergics, but there was a significant decrease in post-exposure IL-13 concentrations for allergics. While surprising, the decrease in IL-5 and IL-13 aligns with the observed drop in blood eosinophils, may support the hypothesis that eosinophils from the peripheral blood may have migrated into the nasal mucosa.

The anti-inflammatory marker, IL-10, is responsible for the downregulation of the immune system following activation to prevent tissue damage and restore homeostasis. Given that IL-10 expression has been found to be negatively correlated with the development and severity of AR, HDM-allergic participants would be expected to have decreased IL-10 serum concentrations (26). Change in IL-10 concentrations does show a decrease for allergics (mean = −0.8188) though not to a significant degree. IL-6 and TNF-α are pro-inflammatory cytokines that play a role in B cell regulation. These cytokines are also stimulated following endotoxin exposure and although HDM extracts typically contain lipopolysaccharide endotoxin among the allergen proteins, the concentration present in the HDM used in the HDM-EEU was within a reasonable limit (27). No significant differences were observed in the change of IL-6 and TNF-α serum concentrations between allergics and non-allergics, though non-allergics had significantly decreased TNF-α concentrations post-exposure in the paired analysis.

These results are variable, which is not unexpected as there is natural variability in individuals' cytokine expressions. Previous studies involving participants with perennial AR showed a significant decrease in IL-4, an increase in IL-5, an increase in IL-6, a decrease in IL-10, and unchanged IL-13 concentrations compared to controls; however, these were evaluated in nasal fluid samples (28). Time of collection may be another reason why the serum cytokine results observed in this cohort differ from what is reported in the literature. The post-exposure blood samples were collected soon after participants completed the 3-h exposure in the HDM-EEU. A longer timespan between exiting the facility and post-exposure blood sample collection may have allowed the localized immune reaction in the nose to better spread systemically into the peripheral blood, as the late-phase AR response is thought to occur within 4–6 h of allergen exposure (29). However, even in nasal fluid following NAC, cytokine expression levels for perennial AR do not appear to be as distinctly changed as for seasonal AR (28).

As the majority of our allergic participants were polysensitized, it is a possible confounding factor that they may have been exposed to other allergens prior to the HDM challenge. Efforts were made to mitigate this, including running the study at the end of grass season, prior to the beginning of ragweed season. However, as HDM is a perennial allergen, participants may have likely been exposed to it outside of the HDM-EEU, such as in their homes. Chronic exposure to perennial allergens may result in decreased sensitivity to allergen so for some allergic participants and this may have impacted the biologic responses to allergen exposure.

These findings establish the applicability of the HDM-EEU for studying mechanisms of HDM-induced AR, as it can produce measurable biological changes in allergic participants. We've shown that SPT wheal sizes confirmed allergic status of participants, for the purpose of this study, and sIgE levels were significantly greater and more variable among the allergic participants in comparison to healthy controls. CBCs and serum cytokine concentrations demonstrate decreased eosinophils and eosinophil-related markers in the peripheral blood post-HDM exposure specifically for allergics.

While AR is not a life-threatening condition, it greatly affects quality of life for patients and their families. Translational clinical models, such as the HDM-EEU, serve to reproduce AR symptoms in a controlled manner and allow for pathophysiological changes upon allergen exposure to be further evaluated.

## Data Availability Statement

The original contributions presented in the study are included in the article/Supplementary Material, further inquiries can be directed to the corresponding author/s.

## Ethics Statement

This study was reviewed and ethics clearance was granted by the Queen's University Health Sciences and Affiliated Teaching Hospitals Research Ethics Board (DMED-19149616). All participants reviewed and provided signed consent prior to study enrolment.

## Author Contributions

AKE developed the protocol, oversaw the study, and ensured critical revision of the manuscript. LH contributed to the conduct the study, conducted the statistical data analyses. SL contributed to the conduct of the study and co-drafted the manuscript with LH. JT contributed to the conduct of the study and edited the manuscript. LS contributed to the development of the study protocol, management of the trial, and revisions to the manuscript. CM was responsible for participant recruitment and revisions to the manuscript. TW was responsible for all operations related to the HDM-EEU and contributed to the manuscript. All authors have read and approved the final manuscript.

## Funding

Self funded by the Kingston Allergy Research Trust. HDM source material provided in kind by ALK-Abeló, Denmark.

## Conflict of Interest

AKE has participated in advisory boards for Abbvie, ALK Abello, AstraZeneca, Aralez, Bausch Health, Circassia Ltd., GSK, LEO Pharma, Merck, Novartis, and Pfizer; has been a speaker for ALK Abello, Aralez, AstraZeneca, CSL Behring, Medexus, Novartis, Mylan, Pfizer, Sanofi, and Takeda. Her institution has received research grants from ALK Abello, Aralez, AstraZeneca, Bayer LLC, Circassia, Green Cross, Merck, Medexus, Pfizer, Novartis, Sanofi, and Regeneron. She has also served as an independent consultant to Bayer LLC and Regeneron. The remaining authors declare that the research was conducted in the absence of any commercial or financial relationships that could be construed as a potential conflict of interest.

## Publisher's Note

All claims expressed in this article are solely those of the authors and do not necessarily represent those of their affiliated organizations, or those of the publisher, the editors and the reviewers. Any product that may be evaluated in this article, or claim that may be made by its manufacturer, is not guaranteed or endorsed by the publisher.
