# Supplementary material for: Biologic Responses to House Dust Mite Exposure in the Environmental Exposure Unit
Source: Front Allergy. 2022 Jan 7;2:807208. doi: 10.3389/falgy.2021.807208 (PMC8974770; doi:10.3389/falgy.2021.807208)
Supplement: Supplementary file 1 [file Data_Sheet_1.docx]

Supplementary Material

# Serum Cytokine Assay

Preparation was required for the magnetic beads, serum matrix, quality controls, Wash Buffer, and standard. 200 µL of Wash Buffer was added into each of the 96 wells of the plate and was mixed on a plate shaker for 10 minutes at room temperature. The buffer was decanted. 50 µL of the prepared standard and quality controls were added to the designated wells, followed by Assay Buffer in all wells. 25 µL of the sample was also put into each well. 25 µL of the beads were included. The plate was sealed and incubated on a plate shaker overnight. The contents of the well were removed and washed with Wash Buffer 3 times before 50 µL of Detection Antibodies were added into each well. The plate was incubated for 1 hour. 50 µL of Streptavidin-Phycoerythrin were added to each well prior to another 30-minute incubation. The contents were removed and washed with Wash Buffer 3 times. 150 µL of Sheath Fluid was added to all wells and the plate was placed on a plate shaker for 5 minutes to resuspend the beads.

# Serum HDM-Specific IgE

The setup, with 40 µL of each sample, was completed as per the standard Phadia^TM^ ImmunoCAP® assay protocol. sIgE from the participants’ serum samples bound to the ImmunoCAP®, which contained the allergens of interest. Wash steps removed non-specific IgE and 50 µL of conjugate was added to each sample to introduce enzyme labelled anti-IgE antibodies to form a complex. The samples were incubated with 50 µL of development solution to convert the enzyme to fluorogenic molecules. 600 µL of stop solution was added to each sample to terminate the reaction.

# Skin Prick Test and Specific IgE in Individual Samples

|  | **Sample Number*** | **Der p** | | **Der f** | |
| --- | --- | --- | --- | --- | --- |
|  |  | **Skin Prick Test** | **Specific IgE** | **Skin Prick Test** | **Specific IgE** |
| Allergics | 1 | 10 | 52.5 | 13 | 62.7 |
|  | 2 | 16 | 3.9 | 16 | 3.37 |
|  | 3 | 9 | 1.24 | 11 | 0.98 |
|  | 4 | 7 | 16.4 | 10 | 13.4 |
|  | 5 | 8 | 1.24 | 7 | 1.35 |
|  | 6 | 9 | 0.69 | 6 | 1.81 |
|  | 7 | 7 | 0.71 | 5 | 0.81 |
|  | 8 | 8 | 0.76 | 8 | 1.06 |
|  | 9 | 9 | 0.51 | 7 | 1.02 |
|  | 10 | 6 | 0.37 | 5 | 0.35 |
|  | 11 | 13 | 0.92 | 10 | 0.92 |
|  | 12 | 6 | 2.64 | 14 | 1.72 |
|  | 13 | 7 | 0.26 | 6 | 0.3 |
|  | 14 | 8 | 0.55 | 9 | 0.64 |
|  | 15 | 11 | 2.01 | 10 | 1.92 |
|  | 16 | 21 | 75.6 | 10 | 78.2 |
|  | 17 | 15 | 1.88 | 20 | 4.69 |
|  | 18 | 7 | 7.77 | 7 | 4.51 |
|  | 19 | 5 | 0.65 | 7 | 0.49 |
|  | 20 | 18 | 2.36 | 14 | 6.15 |
|  | 21 | 14 | 67.7 | 7 | 84.2 |
|  | 22 | 15 | 25 | 7 | 19.4 |
|  | 23 | 15 | 18.4 | 25 | 19 |
|  | 24 | 5 | 3.47 | 7 | 6.29 |
|  | 25 | 17 | 9.04 | 10 | 9.82 |
|  | 26 | 10 | 0.89 | 8 | 1.19 |
|  | 27 | 5 | 0.77 | 7 | 0.64 |
|  | 28 | 10 | 0.52 | 9 | 0.78 |
|  | 29 | 9 | 0.78 | 9 | 0.42 |
|  | 30 | 8 | 0.71 | 6 | 0.69 |
|  | 31 | 9 | 1.83 | 15 | 2.07 |
|  | 32 | 6 | 0.3 | 7 | 0.35 |
|  | 33 | 6 | 1.92 | 7 | 2.09 |
|  | 34 | 5 | 0.13 | 12 | 0.33 |
|  | 35 | 13 | 0.81 | 21 | 0.91 |
|  | 36 | 12 | 1.18 | 10 | 1.03 |
|  | 37 | 9 | 5.48 | 25 | 4.41 |
|  | 38 | 11 | 1.45 | 13 | 1.54 |
|  | 39 | 6 | 21.1 | 13 | 14.9 |
|  | 40 | 16 | 5.42 | 10 | 5.35 |
|  | 41 | 17 | 54.7 | 12 | 48.1 |
|  | 42 | 11 | 14 | 12 | 12.7 |
|  | 43 | 13 | 5.23 | 15 | 7.85 |
|  | 44 | 8 | 56.2 | 11 | 35.7 |
| Non-Allergics | 45 | 0 | 0.05 | 0 | 0.03 |
|  | 46 | 0 | 0.05 | 0 | 0.03 |
|  | 47 | 0 | 0.04 | 0 | 0.04 |
|  | 48 | 0 | 0.02 | 0 | 0.03 |
|  | 49 | 0 | 0.04 | 0 | 0.02 |
|  | 50 | 0 | 0.05 | 0 | 0.05 |
|  | 51 | 0 | 0.1 | 0 | 0.1 |
|  | 52 | 0 | 0.24 | 0 | 0.28 |
|  | 53 | 0 | 0.03 | 0 | 0.02 |
|  | 54 | 0 | 0.04 | 0 | 0.03 |
|  | 55 | 0 | 0.03 | 0 | 0.02 |
|  | * Each sample refers to a study participant. | | | | |

# Participant Demographics

| Characteristics | Modest Target | | Higher Target | |
| --- | --- | --- | --- | --- |
|  | **HDM-Allergic**  **(n=20)** | **Non-Allergic**  **(n=5)** | **HDM-Allergic**  **(n=24)** | **Non-Allergic**  **(n=6)** |
| Mean age, y (SD) | 41.4 (14.6) | 47.6 (12.5) | 45.5 (12.9) | 43.2 (15.0) |
| Women, n (%) | 15 (75.0) | 5 (100.0) | 14 (58.3) | 3 (50.0) |
| Mean weight, kg (SD) | 96.8 (28.5) | 83.7 (10.3) | 93.8 (27.6) | 101.8 (41.5) |
| Mean height, cm (SD) | 166.9 (10.3) | 159.2 (4.2) | 167.0 (11.3) | 171.9 (10.7) |
| Race, n (%)  White  White and Asian  Asian  White & Black or African American | 19 (95.0)  -  1 (5.0)  - | 5 (100.0)  -  -  - | 22 (91.7)  1 (4.2)  -  1 (4.2) | 5 (83.3)  -  1 (16.7)  - |

SD = standard deviation
